# Association Between the Triglyceride-Glucose Index and Acute Myocardial Infarction: A Retrospective Case-Control Study

**DOI:** 10.7759/cureus.101422

**Published:** 2026-01-13

**Authors:** Alfredo Isaí Rojo Mendoza, Jorge Arturo Blanco Olivas

**Affiliations:** 1 Department of Internal Medicine, Hospital General Regional No. 66, Instituto Mexicano del Seguro Social, Ciudad Juárez, MEX

**Keywords:** acute myocardial infarction, cardiovascular risk, coronary artery disease, insulin resistance, metabolic markers, triglyceride-glucose index

## Abstract

Introduction: Acute myocardial infarction (AMI) remains a leading cause of morbidity and mortality worldwide. Metabolic abnormalities, particularly insulin resistance, play a central role in the development of atherosclerosis and coronary artery disease. The triglyceride-glucose (TyG) index has emerged as a simple and accessible surrogate marker of insulin resistance and has been associated with cardiovascular risk in diverse populations. However, data regarding its association with AMI in hospitalized populations in Mexico are limited.

Methods: We conducted an observational, analytical, retrospective, non-paired case-control study including adult patients hospitalized in the cardiology department of a secondary care hospital between January 2023 and December 2024. The cases were patients with a confirmed diagnosis of AMI, while controls were patients hospitalized for non-ischemic conditions during the same period. Demographic and clinical variables were collected from electronic medical records. Fasting plasma glucose and triglyceride levels obtained within the first 48 hours of admission were used to calculate the TyG index. Statistical analyses included receiver operating characteristic (ROC) curve analysis and logistic regression models to estimate crude and adjusted odds ratios (ORs).

Results: A total of 288 patients were included, with 144 cases and 144 controls. Patients with AMI had significantly higher TyG index values compared with controls. The TyG index demonstrated good discriminative performance for AMI, with an area under the ROC curve (AUC) of 0.84. A cutoff value of 8.92 was associated with higher odds of AMI. In multivariable logistic regression analysis, an elevated TyG index remained independently associated with AMI after adjustment for age, sex, hypertension, diabetes mellitus, and dyslipidemia.

Conclusion: An elevated TyG index was independently associated with AMI in this retrospective case-control study. These findings support the potential utility of the TyG index as an accessible metabolic marker associated with increased odds of AMI in hospitalized patients.

## Introduction

Acute myocardial infarction (AMI) remains one of the leading causes of morbidity and mortality worldwide and continues to represent a major public health challenge despite advances in preventive strategies, early diagnosis, and reperfusion therapies [1]. The global burden of AMI is particularly pronounced in low- and middle-income countries, where the prevalence of cardiometabolic risk factors such as diabetes mellitus, dyslipidemia, obesity, and hypertension has increased substantially in recent decades [2,3]. These conditions play a central role in the development and progression of atherosclerosis and significantly increase the risk of acute coronary events.

Insulin resistance is a key pathophysiological mechanism underlying cardiometabolic disease and atherosclerotic cardiovascular disease [4]. However, its direct assessment is not routinely feasible in clinical practice due to methodological complexity and cost. In this context, the triglyceride-glucose (TyG) index, calculated using fasting triglyceride and glucose levels, has emerged as a simple, inexpensive, and reliable surrogate marker of insulin resistance that can be easily obtained from routinely available laboratory parameters [5,6].

Accumulating evidence has demonstrated that elevated TyG values are associated with metabolic syndrome, increased severity of coronary artery disease, major adverse cardiovascular events, and cardiovascular mortality [7-9]. Furthermore, multiple studies have reported a significant association between higher TyG levels and the occurrence, recurrence, and prognosis of AMI, both in patients with and without diabetes mellitus [10-14]. These findings suggest that the TyG index may serve as a useful tool for cardiovascular risk stratification in diverse clinical settings.

Nevertheless, most available data originate from Asian or European populations, while evidence from Latin American countries, particularly Mexico, remains limited. This gap is clinically relevant given the high prevalence of metabolic disorders, the substantial burden of ischemic heart disease, and the documented disparities in AMI outcomes within the Mexican healthcare system [2,3]. Secondary care hospitals within the Instituto Mexicano del Seguro Social (IMSS) provide care to a large population with complex cardiometabolic profiles; however, local data evaluating the association between the TyG index and AMI are scarce.

Therefore, the aim of this study was to evaluate the association between the TyG index and the presence of AMI in patients treated at a secondary care hospital of the IMSS in Ciudad Juárez, Mexico. Identifying the clinical relevance of the TyG index in this context may contribute to improved cardiometabolic risk stratification and support its use as a practical, low-cost tool in routine clinical practice.

## Materials and methods

Study design and setting

We conducted an observational, analytical, retrospective, non-paired case-control study based on the review of medical records. The study was carried out in the cardiology department of Hospital General Regional No. 66, IMSS, a secondary care hospital located in Ciudad Juárez, Mexico. The study period included patients admitted between January 2023 and December 2024.

Study population

The study population consisted of adult patients hospitalized during the study period. Cases were defined as patients with a confirmed diagnosis of AMI, established according to the Fourth Universal Definition of Myocardial Infarction, including clinical presentation, electrocardiographic changes, and elevated cardiac biomarkers [1]. Controls were patients hospitalized during the same period for non-ischemic conditions, without a documented history or diagnosis of AMI.

Patients with incomplete medical records, missing fasting glucose or triglyceride measurements, or conditions that could significantly interfere with lipid or glucose metabolism (such as acute systemic infections, advanced liver disease, or chronic inflammatory conditions) were excluded.

Data collection

Clinical and biochemical data were obtained retrospectively from electronic medical records. Collected variables included demographic characteristics (age and sex), relevant comorbidities (diabetes mellitus, hypertension, and dyslipidemia), and laboratory parameters. 

Fasting plasma glucose and fasting triglyceride levels measured at hospital admission were recorded for all included patients.

TyG index

The TyG index was calculated using the following formula:

$\text{TyG Index} = \ln \left( \frac{\text{Fasting triglycerides (mg/dL)} \times \text{Fasting glucose (mg/dL)}}{2} \right)$

The TyG index was analyzed as a continuous variable and subsequently categorized using an optimal cutoff value derived from receiver operating characteristic (ROC) curve analysis.

Statistical analysis

Continuous variables were assessed for normality using the Shapiro-Wilk test. Variables with non-normal distribution were expressed as median and interquartile range (IQR), while categorical variables were expressed as frequencies and percentages. Comparisons between cases and controls were performed using the Wilcoxon rank-sum test for continuous variables and the Pearson's chi-square test for categorical variables.

ROC curve analysis was used to evaluate the discriminatory capacity of the TyG index for the presence of AMI and to determine the optimal cutoff point. Univariate and multivariate logistic regression analyses were performed to assess the association between the TyG index and AMI, adjusting for clinically relevant confounders. Results were expressed as odds ratios (ORs) with 95% confidence intervals (CIs).

The sample size was estimated a priori for a non-paired case-control study with a 1:1 allocation ratio using a two-sided test for the comparison of two proportions (G*Power v3.1.9.4; Heinrich-Heine-Universität Düsseldorf, Düsseldorf, Germany). An alpha level of 0.05 and 80% statistical power were assumed, with an expected prevalence of elevated TyG in controls of 40% and an anticipated OR of 2.0, corresponding to an estimated exposure proportion of 57.1% in cases. Under these assumptions, the minimum required sample size was 144 participants per group (288 total).

A p-value <0.05 was considered statistically significant. Statistical analyses were performed using Stata/MP 19 (StataCorp LLC, College Station, USA).

Ethical considerations

The study protocol was reviewed and approved by the Local Research and Ethics Committee of IMSS with institutional registration number R-2025-802-121. Given the retrospective nature of the study and the use of de-identified data, informed consent was waived. The study was conducted in accordance with national regulations and the principles of the Declaration of Helsinki.

## Results

Study population

A total of 288 patients treated at Hospital General Regional No. 66 of the IMSS in Ciudad Juárez, Mexico, were included in the analysis. Of these, 144 patients comprised the case group (AMI) and 144 patients served as controls.

The median age of the overall population was 59 years (IQR: 52-66). Both groups were comparable with respect to age and sex distribution, with a predominance of male patients in the total sample. 

Regarding comorbidities, diabetes mellitus and dyslipidemia were significantly more frequent in the case group compared with controls, whereas hypertension was highly prevalent in both groups without reaching statistical significance. Baseline characteristics of the study population are summarized in Table 1.

**Table 1:** Baseline characteristics of the study population Continuous variables were expressed as median and IQR and compared using the Mann-Whitney U test. Categorical variables were expressed as numbers (percentages) and compared using Pearson's chi-square test. IQR: interquartile range

Characteristic	Cases (n = 144)	Controls (n = 144)	Total (N = 288)	p-value
Age in years, median (IQR)	60 (53-66)	58 (51.5-65.5)	59 (52-66)	0.308
Male sex, n (%)	109 (75.7%)	105 (72.9%)	214 (74.3%)	0.59
Hypertension, n (%)	119 (82.6%)	107 (74.3%)	226 (78.5%)	0.085
Diabetes mellitus, n (%)	116 (80.5%)	73 (50.7%)	189 (65.6%)	<0.001
Dyslipidemia, n (%)	89 (61.8%)	34 (23.6%)	123 (42.7%)	<0.001

Primary outcome

**Figure 1:**
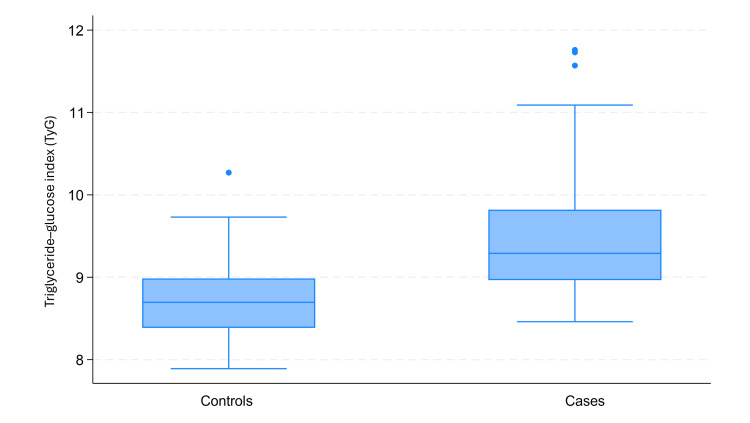
Distribution of the TyG index in cases and controls Box-and-whisker plots showing the distribution of the TyG index in patients with acute myocardial infarction (cases) and controls. The boxes represent the IQR, the horizontal line inside each box indicates the median, and the whiskers represent the minimum and maximum values. Dots indicate outliers. TyG: triglyceride-glucose; IQR: interquartile range

The TyG index differed significantly between study groups. Patients with AMI exhibited significantly higher TyG values compared with the control group (Figure 1).

Median TyG was 9.29 (IQR: 8.96-9.82) in cases and 8.70 (IQR: 8.39-8.98) in controls, with a statistically significant difference between groups (p < 0.001). Because the TyG index did not follow a normal distribution, comparisons were performed using the Mann-Whitney U test.

Bivariate analysis

In bivariate analysis, the presence of AMI was significantly associated with higher TyG values, diabetes mellitus, and dyslipidemia. Hypertension showed a trend toward association but did not reach statistical significance. No significant differences were observed with respect to age or sex distribution between groups.

Detailed results of the bivariate comparisons are shown in Table 2.

**Table 2:** Bivariate comparison between cases and controls Continuous variables were compared using the Mann-Whitney U test, and categorical variables were compared using Pearson’s chi-square test. TyG: triglyceride-glucose

Variable	Statistical test	Test statistic	p-value
Age	Mann-Whitney U	z = −1.02	0.308
TyG index	Mann-Whitney U	z = −9.99	<0.001
Male sex	Chi-square	χ² = 0.291	0.59
Hypertension	Chi-square	χ² = 2.96	0.085
Diabetes mellitus	Chi-square	χ² = 28.46	<0.001
Dyslipidemia	Chi-square	χ² = 42.93	<0.001

Determination of the optimal TyG cutoff value

**Figure 2:**
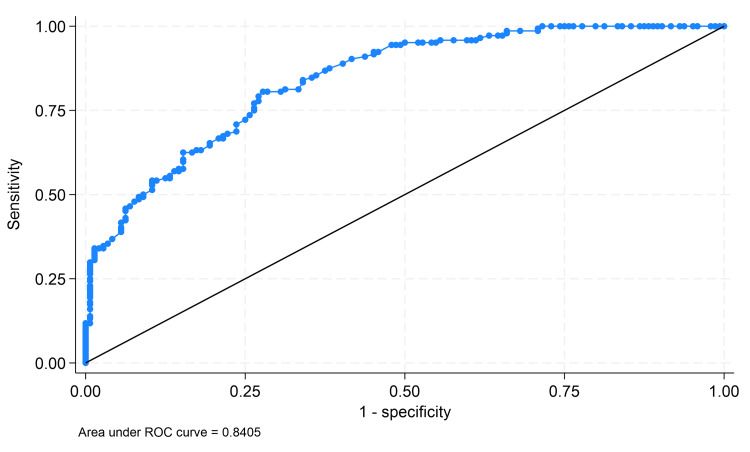
ROC curve of the TyG index for the prediction of acute myocardial infarction ROC curve illustrating the ability of the TyG index to discriminate between patients with and without acute myocardial infarction. The AUC was 0.8405, demonstrating good predictive performance. The diagonal line indicates no discriminative ability. ROC: receiver operating characteristic; AUC: area under the ROC curve; TyG: triglyceride-glucose

ROC curve analysis demonstrated excellent discriminative ability of the TyG index for distinguishing patients with AMI from controls (Figure 2).

The area under the ROC curve (AUC) was 0.8405 (95% CI: 0.7965-0.8846), indicating good diagnostic performance. The optimal cutoff value identified was TyG ≥ 8.92, which yielded a sensitivity of 80.56% and a specificity of 72.22%, representing an adequate balance between both measures.

Association between the TyG index and AMI

Based on ROC analysis, the TyG index was categorized as elevated when values were ≥ 8.92. In bivariate analysis, patients with TyG ≥ 8.92 had a 10.7-fold higher odds of AMI compared with those with lower values (crude OR = 10.78; 95% CI: 6.20-18.50; p < 0.001) (Table 3).

**Table 3:** Association between TyG index ≥ 8.92 and acute myocardial infarction The association between TyG index categories and acute myocardial infarction is presented. A TyG value ≥ 8.92 was significantly associated with the presence of acute myocardial infarction. Crude OR = 10.78 (95% CI, 6.2-18.5); χ² = 80.78; p < 0.001. TyG: triglyceride-glucose; OR: odds ratio; CI: confidence interval

TyG index	Controls (n = 144)	Cases (n = 144)
< 8.92	104 (72.2%)	28 (19.4%)
≥ 8.92	40 (27.8%)	116 (80.6%)

A multivariable logistic regression model was subsequently constructed, adjusting for age, sex, hypertension, diabetes mellitus, and dyslipidemia (Table 4). After adjustment, TyG ≥ 8.92 remained the strongest independent factor associated with AMI (adjusted OR = 5.48; 95% CI: 2.72-11.03; p < 0.001).

**Table 4:** Multivariable logistic regression model for acute myocardial infarction The multivariable logistic regression model adjusted for age, sex, hypertension, diabetes mellitus, and dyslipidemia is presented. Pseudo R² = 0.2439. AUC = 0.8127. OR: odds ratio; CI: confidence interval; TyG: triglyceride-glucose; AUC: area under the ROC curve; ROC: receiver operating characteristic

Variable	Adjusted OR	95% CI	p-value
TyG ≥ 8.92	5.48	2.72-11.03	<0.001
Age	0.996	0.968-1.024	0.783
Male sex	0.84	0.43-1.65	0.611
Hypertension	1.83	0.90-3.73	0.095
Diabetes mellitus	2.15	1.09-4.27	0.028
Dyslipidemia	2.35	1.21-4.55	0.012

In the adjusted model, diabetes mellitus and dyslipidemia were also independently associated with AMI, whereas age, male sex, and hypertension were not statistically significant. The model demonstrated adequate explanatory capacity (pseudo R² = 0.2439) and discrimination (AUC = 0.8127).

Sensitivity analyses using TyG as a continuous variable

To assess the robustness of the association and avoid information loss from dichotomization, the TyG index was additionally analyzed as a continuous variable. Higher TyG values were independently associated with increased odds of AMI (OR per unit increase = 28.03; 95% CI: 9.07-86.57; p < 0.001).

When analyzed as a standardized continuous variable, each one standard deviation (SD) increase in TyG was associated with a significant increase in the odds of AMI (OR per 1 SD = 9.52; 95% CI: 4.44-20.42; p < 0.001), after adjustment for the same covariates.

## Discussion

Principal findings

In this case-control study conducted at a secondary care hospital of the IMSS, the TyG index was significantly associated with the presence of AMI. Patients with AMI exhibited markedly higher TyG values compared with controls, and this difference remained statistically significant after adjustment for major cardiometabolic risk factors. Notably, a TyG cutoff ≥ 8.92 emerged as the strongest independent predictor of AMI in the multivariable model, increasing the odds of infarction more than fivefold.

These findings support the hypothesis that insulin resistance-related metabolic dysregulation plays a central role in the development of acute coronary events and reinforce the clinical relevance of the TyG index as a simple and accessible marker of cardiovascular risk.

TyG index and cardiometabolic risk

Insulin resistance is a key pathophysiological mechanism linking metabolic disorders to atherosclerotic cardiovascular disease. However, direct assessment methods are rarely feasible in routine clinical practice. In this context, the TyG index has gained increasing attention as a reliable surrogate marker derived from fasting triglyceride and glucose levels.

In the present study, TyG values differed significantly between cases and controls, with an AUC of 0.84, indicating good discriminative performance for identifying patients with AMI. This level of accuracy is consistent with previously reported values in international cohorts and supports the utility of TyG as a marker of metabolic risk in acute coronary syndromes [12-14].

Independent association with AMI

After adjustment for age, sex, hypertension, diabetes mellitus, and dyslipidemia, TyG ≥ 8.92 remained the strongest independent factor associated with AMI. Importantly, while diabetes mellitus and dyslipidemia also showed independent associations, traditional risk factors such as age, male sex, and hypertension did not retain statistical significance in the adjusted model.

This finding suggests that the TyG index captures a broader metabolic risk profile that may not be fully reflected by individual clinical diagnoses alone. By integrating both lipid and glucose metabolism, the TyG index may better represent the underlying insulin resistance and proatherogenic milieu that predisposes patients to plaque instability and acute coronary events.

Comparison with previous studies

Multiple studies have demonstrated an association between elevated TyG values and adverse cardiovascular outcomes, including coronary artery disease severity, major adverse cardiovascular events, infarct recurrence, and mortality [5,12,14]. The cutoff identified in our population (TyG ≥ 8.92) falls within the range reported in prior investigations, although variability across studies highlights the influence of population characteristics and clinical context [12-14].

Our findings extend this evidence to a Mexican population treated within the IMSS healthcare system, a setting characterized by a high prevalence of metabolic disorders and a substantial burden of ischemic heart disease. The consistency of the association observed in our study reinforces the external validity of the TyG index across different populations.

Clinical implications

From a clinical perspective, the TyG index offers several advantages. It is inexpensive, easily calculated using routinely available laboratory parameters, and does not require specialized testing. In resource-limited settings, such as many public healthcare institutions in Latin America, TyG may represent a practical tool for cardiovascular risk stratification.

The strong independent association between TyG ≥ 8.92 and AMI suggests that this index could help identify high-risk patients who may benefit from more intensive preventive strategies, closer metabolic control, and targeted cardiovascular surveillance, even in the absence of overt diabetes mellitus.

While the use of hospitalized controls may limit generalizability, this comparison reflects the population in which the TyG index is most likely to be applied in routine clinical practice. Rather than serving as a screening tool for low-risk individuals, the TyG index may be particularly useful for risk stratification among patients with established cardiometabolic risk, supporting its potential role in reinforcing preventive strategies in high-risk settings.

Strengths and limitations

This study has several strengths, including a well-defined case-control design, balanced groups, and comprehensive adjustment for major cardiometabolic confounders. Additionally, the use of routinely collected clinical and laboratory data enhances the applicability of the findings to real-world practice.

However, certain limitations should be acknowledged. The retrospective design precludes causal inference, and residual confounding cannot be entirely excluded. In addition, TyG was calculated using fasting glucose and triglyceride levels obtained during the acute phase of myocardial infarction, when metabolic stress, systemic inflammation, and early pharmacological treatments may influence these parameters. Therefore, TyG values may partially reflect an acute metabolic response rather than pre-event insulin resistance. The single-center nature of the study may also limit generalizability. Although no matching by age or comorbidities was performed, potential confounding was partially mitigated through multivariable logistic regression, adjusting for major cardiometabolic risk factors.

Future directions

Future prospective and multicenter studies are warranted to validate optimal TyG cutoff values in diverse Mexican and Latin American populations and to assess the prognostic value of longitudinal TyG measurements. Additionally, integrating the TyG index into existing cardiovascular risk models may further improve risk prediction and clinical decision-making.

## Conclusions

In this retrospective case-control study conducted at a secondary care hospital of the IMSS, the TyG index was independently associated with the presence of AMI. Patients with AMI exhibited significantly higher TyG values compared with controls, and a cutoff value of TyG ≥ 8.92 demonstrated good discriminative ability and remained the strongest independent predictor of AMI after adjustment for major cardiometabolic risk factors.

Given its simplicity, low cost, and availability from routine laboratory parameters, the TyG index represents a practical tool for cardiometabolic risk assessment in clinical settings with a high burden of metabolic disease. These findings support the potential role of the TyG index as an accessible marker for identifying patients at increased risk of AMI and underscore the need for future prospective and multicenter studies to validate its prognostic value and optimal cutoff points in broader populations.
